# Antibiotic Use in Very Low Birth Weight Neonates After an Antimicrobial Stewardship Program

**DOI:** 10.3390/antibiotics10040411

**Published:** 2021-04-09

**Authors:** Alberto Berardi, Isotta Zinani, Cecilia Rossi, Eugenio Spaggiari, Virginia D’Amico, Greta Toni, Luca Bedetti, Laura Lucaccioni, Lorenzo Iughetti, Licia Lugli

**Affiliations:** 1 Neonatal Intensive Care Unit, University Hospital of Modena and Reggio Emilia, 41121 Modena, Italy; rossi.cecilia@aou.mo.it (C.R.); spaggiari.eugenio@aou.mo.it (E.S.); luca.bedetti@unimore.it (L.B.); lugli.licia@aou.mo.it (L.L.); 2 Pediatric Post-Graduate School, University Hospital of Modena and Reggio Emilia, 41121 Modena, Italy; isotta.zinani@hotmail.it (I.Z.); damicovirgi@gmail.com (V.D.); greta.toni@virgilio.it (G.T.); 3 Ph.D. Program in Clinical and Experimental Medicine, University of Modena and Reggio Emilia, 41121 Modena, Italy; 4 Pediatric Department, University of Modena and Reggio Emilia, 41121 Modena, Italy; lucaccioni.laura@aou.mo.it (L.L.); lorenzo.iughetti@unimore.it (L.I.)

**Keywords:** early onset sepsis, late onset sepsis, antimicrobial stewardship, newborn, very low birth weight neonates

## Abstract

There is insufficient data regarding antimicrobial stewardship (AS) and outcomes of very low birth weight (VLBW) neonates after AS programs. This observational, retrospective study addressed AS and outcomes of VLBW neonates admitted to an Italian level-three center. Two periods were compared: (i) baseline, before AS (January 2011–December 2012) and (ii) intervention, after AS (January 2016–December 2017). Between these two periods, procedures were put in place to inform medical and nursing staff regarding AS. There were 111 and 119 VLBW neonates in the baseline (6744 live births) and in the intervention period (5902 live births), respectively. The number of infants exposed to antibiotics (70%) during the hospital stay did not change, but the total days of therapy (DOT, median 12 vs. 5) and DOT/1000 patient days (302 vs. 215) decreased in the intervention period (*p* < 0.01), as well as the median duration of first antibiotic treatment (144 vs. 48 h, *p* < 0.01). A re-analysis of single cases of culture-proven or culture-negative sepsis failed to demonstrate any association between deaths and a delay or insufficient antibiotic use in the intervention period. In conclusion, AS is feasible in preterm VLBW neonates and antibiotic use can be safely reduced.

## 1. Introduction

Neonatal sepsis is a serious and potentially fatal illness; early diagnosis and prompt treatment is essential to prevent life threatening complications. Antibiotics play a pivotal role in the treatment of neonatal infections and are the most commonly used drugs in the neonatal intensive care unit (NICU) [1]. Both early onset sepsis (EOS) and late onset sepsis (LOS), which present prior to or after 72 h of life, respectively, are much more frequent in preterm neonates with very low birth weight (VLBW) compared to full term neonates, and the risk of infectious mortality is exceedingly high. Therefore, it is not surprising that more than 75% of VLBW neonates are given antibiotics for the risk of EOS, often for prolonged periods [2,3]. However, early and reliable markers of sepsis are unavailable, whereas the potential negative impacts of empiric antibiotic administration itself in the immediate neonatal period are being evaluated. Perinatal antibiotics can cause intestinal dysbiosis, which has been associated with short- and long-term diseases [4,5,6]. Newborns receiving prolonged antibiotic therapies (7–10 days) have a lower diversity of microbiota in the first days of life, whereas neonates exposed to short antibiotic therapies have a more uniform distribution of the various genera [4]. Consequently, prolonged early empiric antibiotic administration in preterm neonates has been associated with an increased risk of necrotizing enterocolitis (NEC) [7], LOS and death, as well as the risk of invasive fungal infections [8], or selecting multidrug-resistant pathogens [9,10]. Finally, antibiotic exposure increases the risks of acute drug toxicities, the costs, and potential unintended consequences of escalated monitoring [11,12].

Antimicrobial stewardship (AS) refers to a series of coordinated interventions, which aim to minimize the unintended consequences of antimicrobial use (including toxicity and the emergence of antimicrobial resistance), while maintaining the safety of the patient [13]. AS programs guide the optimal choice of drugs, dosage, duration of therapy, and route of administration [14].

There are insufficient studies regarding AS in neonates. Impact and safety of AS programs for neonates are not clearly defined, particularly in neonates with a lower birth weight [15], who are at increased risk of sepsis. The aim of this study was to assess changes of antibiotic use in a cohort of VLBW neonates admitted to an Italian level-three center before and after an AS program was introduced. Furthermore, medical records of cases with culture-proven and culture-negative sepsis and sepsis-related deaths were reviewed in order to assess the outcomes of neonates after introducing the AS protocol.

## 2. Results

A total of 230 VLBW neonates (111 in the baseline and 119 in the intervention period) were enrolled. A few neonates were excluded from the study because of missing data on antibiotic treatments: eight neonates in the baseline (late admission from another center *n* = 2; transfer to another hospital prior to discharge, *n* = 6) and two neonates in the intervention period (late admission from another center *n* = 1; transfer to another hospital prior to discharge, *n* = 1).

Table 1 compares the remaining 230 VLBW neonates enrolled in the study and the antibiotic treatments in both periods. At some point during their hospital stay, antibiotics were given for shorter or longer periods of time to 82 (74%) out of the 111 (baseline) and to 84 (70%) out of the 119 (intervention, *p* = 0.68) VLBW neonates admitted to the hospital. During the intervention period, 48 h rule-out courses of first antibiotic treatment increased (antibiotic treatments lasting ≤ 72 h are shown in the footnote) and the median duration of first antibiotic treatment decreased. Furthermore, in the intervention period the total days of treatment (DOT) and DOT/1000 patient days decreased. There was a 29% reduction in DOT/1000 patient-days.

**Figure 1:**
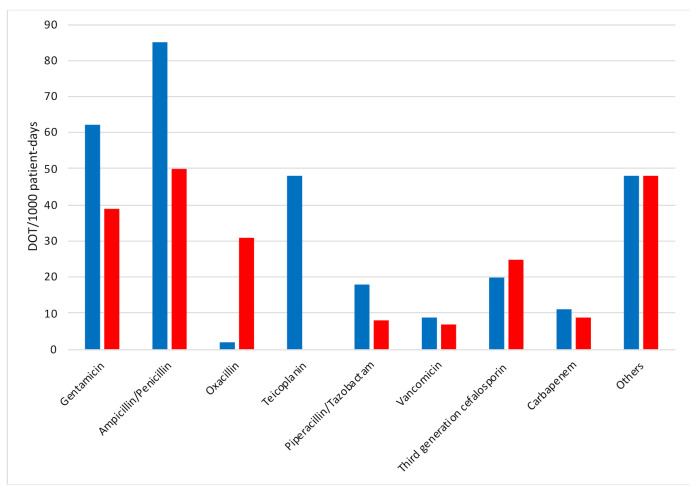
Antibiotic use by drug at baseline and in the intervention period. Baseline, blue columns; intervention period, red columns. DOT, days of therapy.

Figure 1 shows each drug according to the DOT/1000 patient-days at baseline and in the intervention period. There was a 41% decrease in the use of ampicillin and penicillin (DOT/1000 patient days = 85 at baseline vs. DOT/1000 patient days = 50 in the intervention period, *p* < 0.01) and a 37% decrease in the use of gentamicin (DOT/1000 patient days = 62 at baseline vs DOT/1000 patient days = 39 in the intervention period, *p* < 0.02). Furthermore, use of teicoplanin decreased (DOT/1000 patient days = 48 at baseline and 0 in the intervention period, *p* < 0.01) and oxacillin use increased (DOT/1000 patient days = 1 at baseline and 31 in the intervention period, *p* < 0.01). The use of piperacillin and tazobactam decreased (DOT/1000 patient days = 18 at baseline and 8 in the intervention period, *p* < 0.05). There was no significant change in the use of vancomycin, third generation cephalosporin, and carbapenem between the baseline and intervention periods.

### 2.1. Antibiotic Treatments According to Birth Weight

Table 2 compares neonates and antibiotic treatments in both periods according to lower (<1000 g) or higher (≥1000 g) birth weight. Median duration of first antibiotic treatment, DOT, and DOT/1000 patient days were all significantly reduced in the intervention period, both in neonates with a birth weight under and over 1000 g, while short courses of antibiotic treatments increased. Compared to neonates with a birth weight ≥ 1000 g, during the intervention period neonates with a birth weight under 1000 g had a longer duration of the first antibiotic treatment (median 72 vs 48 h, *p* < 0.01), and higher median days of therapy (11 vs. 3 days, *p* < 0.01).

### 2.2. Neonates with A Low Risk of EOS

A sub-analysis was carried out in neonates with a low risk of sepsis. Table 3 compares neonates and antibiotic treatments at baseline and in the intervention period. There was a median lower gestational age and Apgar score and a higher rate of intrapartum antibiotic prophylaxis (IAP) in the intervention period. Furthermore, comparable to what was already observed in all VLBW neonates, those with a low risk of EOS during the intervention period had lower DOT and DOT/1000 patient days and the number of 48 h rule-out courses increased. The decrease in DOT/1000 patient days was 42%. Neonatal complications, deaths, and re-institution of antibiotics did not differ between the two periods.

### 2.3. Neonatal Complications, Deaths and Reinstitution of Antibiotics

Table 4 compares sepsis, necrotizing enterocolitis, and reinstitution of antibiotics at baseline and in the intervention period. Culture-negative sepsis decreased significantly in the intervention period.

Case fatalities (all causes) within 72 h of birth were six at baseline and eight in the intervention period. All cases were given broad-spectrum antibiotics from birth to death. Case fatalities (all causes) after 72 h of life were five at baseline and nine in the intervention period. There were four case fatalities due to LOS (one at baseline and three in the intervention period). A re-analysis of single cases of culture-proven or culture-negative sepsis failed to demonstrate any association between the deaths and a delay or insufficient antibiotic use. In both periods, no deaths were due to culture-proven sepsis that presented within 14 days after discontinuing a previous antibiotic treatment. One death at baseline was due to a culture-negative sepsis. Finally, two neonates (one at baseline and one in the intervention period) died because of LOS presenting at three and four days of life, respectively. These newborns were not given antibiotics at birth because of a cesarean section before labor with intact membranes.

## 3. Discussion

Recently, safe strategies have allowed a significant reduction of unnecessary antibiotic use in apparently healthy full-term newborns at risk of EOS through quantitative risk estimates and serial physical examinations [16,17]. In contrast, few studies provide indications for reducing the use of antibiotics in preterm neonates [18]. In fact, preterm neonates, particularly those with a VLBW, have a much higher risk of developing serious infections, as the symptoms at the onset are often ambiguous, and a continuous clinical evaluation is essential to decide whether to start or to discontinue antibiotic therapies. The reduction of unnecessary or redundant antimicrobial therapies in such neonates is therefore a challenge.

In the current study, the total number of infants exposed to antibiotics (70%) during the entire period of hospitalization did not change. This is not surprising, since VLBW infants are a small but high-risk infectious population, in which the use of antibiotics, albeit by short courses, is common. However, the antibiotic use decreased significantly, and DOT/1000 patient days were reduced by 29%. The decline in antibiotic use is comparable to the 27% observed in a recent prospective study which, however, focused on the AS strategy for neonates with a higher gestational age (less than 35 weeks of gestation) admitted to a US NICU [19]. The study showed a decrease in antibiotic use by means of a prospective audit, targeted stewardship interventions, and electronic “hard stops” after 48 h for empiric antibiotic treatment. Furthermore, it was found that the reduction of antimicrobial therapies was not associated with lower safety for neonates. Similarly, Kitano et al. developed a protocol for AS in VLBW neonates in Japan [18]. In a retrospective cohort study, they found a 76% decrease in DOT/1000 patient days, whereas neonates receiving any antibiotic therapy decreased from 55% to 21%, although there were few neonates with extremely low birth weight. The authors ascribed the success to the criteria for initiating and stopping antimicrobial treatment, to the microbiological reporting of blood culture results over the weekend, and to stopping antimicrobial ordering for the next day [18]. However, the impact of AS is not well defined in infants, especially in those with lower gestational age [19,20,21,22]. In a study conducted in the US, 118 (before the AS program) and 282 (after the AS program) extremely low birth weight infants were compared [20]. The authors reported only a slight reduction in the number of days on antibiotic therapy.

In this current study, the decrease in antimicrobial therapies in the intervention period was mostly driven by the reduction of the prolonged antibiotic treatments at birth. This decline was more apparent in neonates with a higher birth weight and occurred without any significant increase in re-institution of antibiotic therapies. Although the rate of short antibiotic courses was only 44% in the intervention period (likely because the intervention was close to a training period), the clinicians’ awareness regarding the safety of an early discontinuation of antimicrobials was reached gradually by the staff. Indeed, during the intervention period most neonates (59%) undergoing a prolonged antibiotic course actually received antibiotics for ≤72 h, and a further reduction in the duration of antibiotic courses continued even after the study period. However, the vast majority of 48 h rule-out courses at birth were unnecessary. It remains a challenge for future studies to investigate and identify the few infants at very high risk of EOS that need to be treated with antibiotics. Furthermore, some glycopeptides (e.g., teicoplanin) not recommended by AS programs were fully replaced, while reduced antibiotic use was particularly evident for some drugs (e.g., ampicillin and gentamicin); however, the use of third generation cephalosporins and vancomycin was not reduced. The unchanged use of vancomycin was not unexpected, since even in the baseline period the first-line antibiotic for LOS was not vancomycin, but rather teicoplanin, which was fully replaced by oxacillin during the intervention period. Finally, because culture-negative sepsis by definition requires an antibiotic treatment of at least five days, and because some antibiotic courses during the intervention became shorter, the number of cases who met the definition of culture-negative sepsis obviously decreased (from 22% to 11%).

Each case of culture-proven or culture-negative sepsis was also re-analyzed; in fact, one of the major concerns of clinicians regarding AS is that giving shorter antibiotic treatments at birth or narrowing the antibiotic spectrum may be associated with an increased risk of infectious relapses and neonatal deaths. We failed to demonstrate any association between deaths and insufficient antibiotic use. However, two neonates with a low risk of EOS died in the baseline and in the intervention period because of a very early LOS (presenting at three and four days of life, respectively). Both were untreated at birth because of a low risk of EOS, as also recently suggested by others [11,23]. However, developmental changes in organ function, which begin immediately upon delivery and support required at birth for reasons unrelated to the infectious process, may make the symptoms of LOS less obvious, particularly in the first days of life. It is not known whether these deaths were due to a delay in administering antibiotics, however caution and close daily vigilance of the clinical condition of the newborn are mandatory when antibiotics are not given at birth in VLBW infants with a low risk of EOS.

This study has some potential limitations. Firstly, our results may not be generalizable to other settings with different personnel, structures, and patient mixes (e.g., settings where most infants are out-born). Furthermore, there is a limitation inherent to retrospective studies with historical comparison. From the baseline to the intervention period additional changes in the therapeutic management of VLBWs (such as improved care) may have occurred. However, the time interval between the two periods in the study is very close, making it unlikely that relevant additional changes had occurred. In addition, although some large studies have shown a reduction in LOS after AS programs [14], we were not able to confirm such a reduction; this is likely due to the small sample size in this study. Finally, due to the retrospective design of the study, we were unable to assess whether the diversity of the gut microbiota had improved, or to evaluate in detail a reduced colonization with multidrug-resistant pathogens, both of which are likely consequences of a reduced use of antibiotics [10].

## 4. Materials and Methods

### 4.1. Study Design

This observational, retrospective study was carried out in the NICU of the University Hospital of Modena, Italy; this is a high-volume level-three facility, with inborn neonates accounting for most admissions. The NICU contains 20 cots, receives 450 admissions per year, and the medical staff consists of 12 physicians. The study project was approved by the local ethics committee (Protocol AOU 0002163/19). The study concerns VLBW neonates, who were admitted to the NICU during two periods: (i) baseline (before AS), from 1st January 2011 to 31st December 2012 (live births *n* = 6744) and (ii) intervention (after AS was implemented), from 1st January 2016 to 31st December 2017 (live births *n* = 5902). Between these two periods, procedures were put in place to inform the medical and nursing staff of the NICU regarding the AS. A review of the EOS and LOS cases and antimicrobial susceptibility of pathogens was initially launched in the entire region [24,25]. Subsequently, multidisciplinary meetings were planned (in 2014 and 2015) among the regional centers for the prevention of neonatal infections and the establishment of an AS program by means of a literature review. Finally, training courses for medical and nursing staff and clinical audits were carried out, and a protocol for the management of newborns was issued [26]. Algorithms for guiding the use of antibiotics were created and the empirical use of antibiotics was revised: broad-spectrum antibiotics (ampicillin and penicillin plus gentamicin for EOS; oxacillin plus an aminoglycoside for LOS) were discontinued within 48 h (to rule out sepsis) in the absence of evidence of sepsis or focal bacterial infection (e.g., pneumonia, meningitis). Sterile cultures, improvement in clinical symptoms, and marked reduction in inflammatory markers (namely, C-reactive protein for EOS, and procalcitonin for LOS) were used as a guide for discontinuing antibiotics. A treatment duration of 5–7 days was recommended for suspected pneumonia or culture-negative sepsis [14,27]. The empirical use of glycopeptides (teicoplanin or vancomycin) was replaced by semi-synthetic penicillin (oxacillin); third generation cephalosporins were administered for suspected meningitis and carbapenems only for multidrug-resistant pathogens. After pathogen isolation, broad-spectrum antibiotics were replaced by narrow-spectrum antibiotics, based on known antimicrobial susceptibility [14,19]. The primary outcome measure was to assess whether changes in antibiotic use occurred between the baseline and the intervention period. As a secondary outcome, we assessed whether there was any clinical worsening due to the change in antibiotic use strategy.

### 4.2. Exclusion Criteria

Infants with an uncertain history of antibiotic use were excluded (e.g., neonates admitted to our center). Among these were newborns that were referred from another hospital or transferred to another center, since it was difficult to assess a possible re-institution of antibiotic therapies. Neonates who were given topical antibiotics were also excluded from the analysis.

### 4.3. Data Collection

Data were collected retrospectively by accessing the following sources: Vermont Oxford Network (VON) database and the NICU computerized medical records (Metavision Suite, iMDSOFT, version 5.40.44, Israel). The following maternal and neonatal characteristics were evaluated: intrapartum antibiotic prophylaxis (IAP) administration, mode of delivery, group B streptococcus antenatal screening [28], risk factors for EOS, gender, gestational age, birth weight, APGAR score at the 5th minute, CRIB (clinical risk index for babies) score, antenatal steroids, placement of central venous line (site and duration), invasive mechanical ventilation, necrotizing enterocolitis (NEC, ≥ Bell’s stage 2) [7], blood and cerebrospinal fluid cultures, infecting organisms, clinical symptoms, sepsis (culture-proven, culture-negative, and sepsis due to coagulase-negative staphylococci), mortality, and length of hospital stay. Data were obtained from computerized records by surveillance officers using a standardized form.

### 4.4. Data Relating to Antibiotic Therapies

The following data were recorded: the timing of the initiation of the first antibiotic treatment and its duration, the drug used as the first course and any reinstitution and, finally, the overall duration of antibiotic therapies during hospitalization.

### 4.5. Definitions

Very low birth weight (VLBW): neonates with a birth weight under 1500 g.

Adequate intrapartum antibiotic prophylaxis (IAP): antibiotics (penicillin, ampicillin or cefazolin) administered ≥4 h prior to delivery [29].

CRIB score (Clinical Risk Index for Babies): neonatal mortality risk estimate score based on variables such as birth weight, gestational age, presence of congenital malformations, maximum negative base excess (BE) in the first 12 h, minimum and maximum oxygen fraction (FiO2) administered in the first 12 h [30].

EOS or LOS: clinical sign of sepsis and yield of a pathogen from blood or cerebrospinal fluid prior to (EOS) or after 72 h of life (LOS).

Coagulase-negative staphylococci (CoNS) were included in the list of pathogens causing sepsis if infants had clinical signs of sepsis, 2 positive blood cultures (collected within 48 h) or antibiotics (glycopeptide or semisynthetic penicillin) were given for ≥5 days [31,32,33].

Culture-negative sepsis: sterile cultures and clinical signs or abnormal laboratory values that could be consistent with sepsis in a neonate who received ≥5 days of antibiotics [34].

Low risk of EOS: neonates born before labor and with an intact membrane, delivered for maternal reasons (e.g., hypertensive disorders), without a clinical or histological diagnosis of chorioamnionitis.

Sepsis-related death: death occurring within 7 days from the positive blood culture or clearly related to complications due to sepsis [25].

First antibiotic treatment: indicates the first treatment during the hospital stay, whether at birth or in the following days.

48 h rule-out course: antibiotic administration for 48 h in neonates with clinical signs of sepsis or abnormal laboratory test results and sterile cultures.

Reinstitution of an antibiotic treatment: a new antibiotic course administered within 14 days after discontinuing (for at least 24 h) the previous course [19]. For each case, the reason for the reinstitution of the treatment was assessed (culture-proven sepsis, culture-negative sepsis, abnormal laboratory test results in apparently healthy neonates, surgical prophylaxis, unknown reasons).

### 4.6. Statistical Analyses

Analyses were performed using STATA/SE 14.2 (StataCorp, Lakeway, TX, USA) and MedCalc^®^ version 9.3 (MedCalc Software, Ostend, Belgium).

For descriptive data comparisons, the Mann–Whitney test was used for non-parametric continuous data and the χ2 test or Fisher exact test for categorical data. Non-parametric continuous variables are summarized as medians with quartiles (25th and 75th percentiles). Categorical variables are presented as percentages. The threshold for statistical significance was *p* < 0.05 for 2-sided tests.

The use of antibiotics was calculated as follows:

- Days of therapy (DOT): number of doses of antibiotic multiplied by the time interval between doses then divided by 24 h [35].
- DOT/1000 days of hospitalization-patient: DOT divided by the total duration of hospitalization of all patients then multiplied by 1000 [35].

## 5. Conclusions

This study shows that AS is feasible in preterm VLBW neonates and antibiotic use can be safely reduced. Nevertheless, close monitoring of antibiotic therapies and careful and continuous vigilance of neonatal outcomes are necessary.

## Figures and Tables

**Table 1:** Comparison of demographics, risk factors for early-onset sepsis, additional characteristics of very low birthweight (VLBW) neonates, and antibiotic use at baseline and in the intervention period.

Variables	Baseline (*n* = 111)	Intervention (*n* = 119)	*p*
Prenatal steroids, *n* (%)	96 (86)	107 (90)	0.42
Maternal indication for delivery, *n* (%)	28 (25)	39 (33)	0.48
Histological chorioamnionitis, *n* (%)	29 (26)	31 (26)	0.99
Twin birth, *n* (%)	28 (25)	31 (26)	0.99
Prolonged membrane rupture (≥ 18 h), *n* (%)	29 (26)	34 (29)	0.79
Maternal fever during labor (> 38 °C), *n* (%)	3 (3)	6 (5)	0.57
Intrapartum antibiotic prophylaxis, *n* (%) No Adequate Inadequate	68 (61) 30 (27) 7 (6)	55 (46) 48 (40) 10 (8)	0.06
Mode of delivery, *n* (%) Vaginal CS in labor or with membrane rupture CS before labor and with intact membranes	21 (19) 30 (27) 60 (54)	25 (21) 25 (21) 69 (58)	0.56
Male gender, *n* (%)	61 (55)	67 (56)	0.94
Gestational age, weeks, median (IQR)	29 (26–31)	29 (26–31)	0.88
Birth weight, g, median (IQR)	1146 (857–1346)	1109 (851–1398)	0.85
Apgar score at the 5th minute, median (IQR)	8 (7–9)	8 (6–9)	0.03
CRIB score, median (IQR)	1 (1–4)	1 (0–4)	0.27
Median length of stay, days (IQR)	47 (29–75)	46 (28–71)	0.99
First antibiotic treatment Total, *n* (%) † 48-h rule-out course, *n* (%) § Median duration, hours (IQR)	82 (74) 3 (4) 168 (120–192)	84 (71) 37 (44) 72 (48–72)	0.68 <0.01 <0.01
Days of therapy Total Median (IQR)	1738 12 (0–23)	1357 5 (0–16)	<0.01 <0.01
Days of therapy/1000 patient-days	302	215	< 0.01

CRIB, clinical risk index for babies; CS, cesarean section; IQR, interquartile range. Indications of intrapartum antibiotic prophylaxis are: maternal group B streptococcus colonization, preterm birth, group B streptococcus bacteriuria identified during the current pregnancy, previous infant with group B streptococcus infection, membrane rupture ≥ 18 h, maternal temperature ≥ 38 °C during labor. † Neonates who were given antibiotics prior to 72 h of life were 78 (95%) at baseline and 74 (88%) in the intervention period. § percent rates were calculated only on neonates who were given antibiotics. The number of neonates undergoing an antibiotic treatment lasting ≤ 72 h was 6 (7%) at baseline and 65 (77%) in the intervention period.

**Table 2:** Length of stay, antibiotic use, sepsis, necrotizing enterocolitis, and reinstitutions of antibiotic treatments in both periods according to birth weight.

Variables	Birth Weight < 1000 g, Baseline(*n* = 44)	Birth Weight < 1000 g, Intervention (*n* = 51)	*p*	Birth Weight 1000–1500 g, Baseline (*n* = 67)	Birth Weight 1000–1500 g, Intervention (*n* = 68)	*p*
Median length of stay, days (IQR)	73 (51–87)	71 (21–96)	0.86	37 (29–53)	42 (28–56)	0.76
First antibiotic treatment Total, *n* (%) 48-h rule-out course, *n* (%) § Median duration, hours (IQR)	39 (89) 2 (5) 168 (126–192)	46 (90) 16 (35) 72 (48–96)	0.93 <0.01 <0.01	43 (64) 1 (2) 168 (126–192)	38 (56) 21 (55) 48 (48–72)	0.42 <0.01 <0.01
Days of therapy Total Median (IQR)	1056 22 (12–36)	893 11 (5–25)	0.01 0.01	682 8 (0–15)	454 3 (0–5)	0.01 0.01
Days of therapy/1000 patient-days	367	266	<0.01	238	154	<0.01
Early-onset sepsis, *n* (%)	3 (7)	3 (6)	0.79	0 (0)	1 (1)	0.99
Late-onset sepsis, *n* (%)	15 (34)	13 (25)	0.36	5 (7)	4 (6)	0.71
Culture-negative sepsis, *n* (%)	15 (34)	9 (18)	0.07	9 (13)	4 (6)	0.14
Necrotizing enterocolitis, *n* (%)	0 (0)	3 (6)	0.30	1 (1)	1 (1)	0.99
Reinstitution of an antibiotic treatment, *n* (%)	16 (36)	19 (37)	0.93	10 (15)	4 (6)	0.08

IQR, interquartile range. § percent rates were calculated only on neonates who were given antibiotics. Sepsis due to coagulase-negative staphylococci (CoNS) were six at baseline and two in the intervention period (extremely low birth weight neonates) or two at baseline and zero in the intervention period (very low birth weight neonates).

**Table 3:** Neonates with a low risk of early-onset sepsis and antibiotic treatments at baseline and in the intervention period.

Variables	Baseline (*n* = 26)	Intervention (*n* = 34)	*p*
Male gender, *n* (%)	12 (46)	18 (53)	0.79
Gestational age, weeks, median (IQR)	31 (29–32)	30 (29–31)	0.04
Birth weight, g, median (IQR)	1320 (1076–1456)	1220 (990–1445)	0.32
Apgar score at the 5th min, median (IQR)	9 (7–10)	8 (7–9)	0.05
CRIB score, median (IQR)	1 (0–1)	1 (0–4)	0.60
Twins, *n* (%)	4 (15)	5 (15)	0.77
Maternal fever in labor (> 38 °C), *n* (%)	0 (0)	1 (3)	0.89
IAP, *n* (%) No Adequate Inadequate	25 (96) 0 (0) 1 (4)	27 (79) 6 (18) 1 (3)	0.04
Prenatal steroids, *n* (%)	22 (85)	31 (91)	0.43
Median length of stay, days (IQR)	47 (38–57)	46 (42–57)	0.99
First antibiotic treatment Total, *n* (%) 48-h rule-out course, *n* (%) § Median duration, hours (IQR) §	13 (50) 0 (0) 144 (96–168)	16 (48) 14 (88) 72 (48–72)	0.97 <0.01 <0.01
Days of therapy Total Median (IQR)	233 2 (0–15)	190 0 (0–6)	<0.01 <0.01
Days of therapy/1000 patient-days	194	113	<0.01

CRIB, clinical risk index for babies. IAP, intrapartum antibiotic prophylaxis. IQR, interquartile range. Indications of intrapartum antibiotic prophylaxis are: maternal group B streptococcus colonization, preterm birth, group B streptococcus bacteriuria identified during the current pregnancy, previous infant with group B streptococcus infection, membrane rupture ≥ 18 h, maternal temperature ≥ 38 °C during labor. § percent rates and median duration were calculated only on neonates who were given antibiotics.

**Table 4:** Sepsis, necrotizing enterocolitis, and reinstitution of antibiotic treatments at baseline and in the intervention period.

Variables	Baseline (*n* = 111)	Intervention (*n* = 119)	*p*
Early-onset sepsis, *n* (%)	3 (3)	4 (3)	0.93
Late-onset sepsis, *n* (%)	20 (18)	17 (14)	0.44
Culture-negative sepsis, *n* (%)	24 (22)	13 (11)	0.04
Necrotizing enterocolitis, *n* (%) †	1 (1)	4 (3)	0.20
Reinstitution of an antibiotic treatment, *n* (%)	26 (23)	23 (19)	0.55
Reasons for the reinstitution of an antibiotic treatment, *n* (%) § Culture-proven sepsis Suspect of sepsis Surgical prophylaxis	5 (19) 17 (65) 4 (15)	7 (30) 14 (61) 2 (9)	0.56 0.98 0.78
Total case fatalities, *n* (%)	11 (10)	17 (14)	0.41

Sepsis due to coagulase-negative staphylococci were eight at baseline and two in the intervention period. † Bell’s stage ≥ 2. § percent rates were calculated only on the total number of antibiotic treatments re-instituted.

## Data Availability

De-identified individual participant data presented in this study are available on request from the corresponding author. The data are not publicly available due to the need for use in further research.
